# Unilateral Tinnitus: Changes in Connectivity and Response Lateralization Measured with fMRI

**DOI:** 10.1371/journal.pone.0110704

**Published:** 2014-10-20

**Authors:** Cornelis P. Lanting, Emile de Kleine, Dave R. M. Langers, Pim van Dijk

**Affiliations:** 1 Department of Otorhinolaryngology/Head and Neck Surgery, University Medical Center Groningen, University of Groningen, Groningen, Netherlands; 2 Graduate School of Medical Sciences (Research School of Behavioural and Cognitive Neurosciences), University of Groningen, Groningen, Netherlands; 3 National Institute for Health Research, Nottingham Hearing Biomedical Research Unit, School of Clinical Sciences, University of Nottingham, Queen’s Medical Centre, Nottingham, United Kingdom; University of Regensburg, Germany

## Abstract

Tinnitus is a percept of sound that is not related to an acoustic source outside the body. For many forms of tinnitus, mechanisms in the central nervous system are believed to play a role in the pathology. In this work we specifically assessed possible neural correlates of unilateral tinnitus. Functional magnetic resonance imaging (fMRI) was used to investigate differences in sound-evoked neural activity between controls, subjects with left-sided tinnitus, and subjects with right-sided tinnitus. We assessed connectivity patterns between auditory nuclei and the lateralization of the sound-evoked responses. Interestingly, these response characteristics did not relate to the laterality of tinnitus. The lateralization for left- or right ear stimuli, as expressed in a lateralization index, was considerably smaller in subjects with tinnitus compared to that in controls, reaching significance in the right primary auditory cortex (PAC) and the right inferior colliculus (IC). Reduced functional connectivity between the brainstem and the cortex was observed in subjects with tinnitus. These differences are consistent with two existing models that relate tinnitus to i) changes in the corticothalamic feedback loops or ii) reduced inhibitory effectiveness between the limbic system and the thalamus. The vermis of the cerebellum also responded to monaural sound in subjects with unilateral tinnitus. In contrast, no cerebellar response was observed in control subjects. This suggests the involvement of the vermis of the cerebellum in unilateral tinnitus.

## Introduction

Subjective tinnitus is a prevalent hearing disorder that is characterized by an auditory sensation in the absence of an external acoustic stimulus. Presumably, hearing loss results in the sensory deprivation of neurons that are tuned to the affected frequencies. In an effort to restore their reduced activity back to normal levels, neurons may change the strength, or gain, of the existing connections or initiate new connections [1]. As a result, spontaneous firing rates (SFR) of neurons in the auditory system may increase [2], [3].

In addition, neural synchrony may also increase as a consequence of neurons responding to the same limited amount of sensory input [2], [4]. Normally a driving stimulus, i.e. a sound source, causes a time-locked elevated firing rate that is synchronous across many neurons. Therefore, when as a consequence of hearing loss and corresponding homeostatic changes in the firing pattern of neurons, both spontaneous activity and synchrony are elevated, it can be perceived as the presence of a sound in the absence of a true sound source [5], [6].

Since spontaneous activity elevation and synchrony cannot be measured using fMRI, other paradigms have been used to study tinnitus [7], [8]. Measuring changes in hemodynamics following the response to a sound in patients with unilateral tinnitus showed an increased sound-evoked response in the inferior colliculus (IC) in patients compared to controls [9], [10]. Recently, however, is was shown that this increased response may have been associated with hyperacusis - a reduced tolerance to loud sounds and commonly described by tinnitus sufferers - rather than with tinnitus [11].

In addition to the changes in firing rate, synchrony, and increased sound-evoked responses, there may be other, more subtle changes. One of these changes may relate to the perceived lateralization of tinnitus. It is conceivable that if tinnitus is perceived strongly lateralized, differences in the sound-evoked activity to monaural stimuli can be observed. Normally, the lateralization to monaural sound is mostly contralateral; evoked responses in the auditory pathway tend to be stronger to contralateral than to ipsilateral stimuli, with a notable exception being the cochlear nucleus, which receives only input from the ipsilateral auditory nerve. If unilateral tinnitus corresponds to reduced or increased lateralized activity along the auditory pathway, it could thus affect the normal lateralization to sound. The hypothesis here is that in patients with unilateral tinnitus the normal lateralization to sound has changed. By using monaural stimuli and measuring the lateralization to sound stimuli, we are able to assess changes related to unilateral tinnitus.

Further, it may be the case that tinnitus corresponds to changes in connectivity patterns between nuclei in the auditory pathway and other non-auditory systems. The auditory part of the thalamus seems to play a specific role in the perception of tinnitus. In a recent model, tinnitus results from impaired inhibitory connections from limbic regions to the auditory thalamus [12], [13]. In this model, tinnitus (originating from e.g. homeostatic changes) would be inhibited after a short while due to feedback connections from limbic areas. If however limbic regions are compromised, this inhibition mechanism that would normally ‘tune out the tinnitus’ breaks down, and chronic tinnitus results. It might thus be the case that due to changes in this corticothalamic feedback-loop the thalamus receives less inhibition, which in turn may results in changes in the functional connectivity between the auditory brainstem and auditory cortex.

In a different model of tinnitus generation the thalamus also plays a major role. Specifically, thalamocortical rhythms that naturally occur in the brain, relating to e.g. sleep and consciousness [14], may be affected by deafferentation due to e.g. hearing loss in the case of tinnitus. It is thought that, as a consequence of such reduced input to the thalamus or a protracted functioning of the thalamus, the normal rhythms of the thalamocortical loop change to an increased large-scale, slow-rate oscillatory coherent theta (4–8 Hz) activity, in turn reducing lateral inhibition and disinhibiting more high-frequent gamma (30–70 Hz) oscillations [15]. As a consequence, we hypothesize that both models lead to changes in the normal connectivity-patterns between the brainstem, the thalamus, and the cortex.

In a recent study of tinnitus in subjects with moderate sensory hearing loss, indeed a reduction in functional connectivity was observed between the brainstem and cortex [16]. The goal of this study was to measure this in a group of participants with near-normal hearing. We therefore I) measure sound-evoked response levels in the auditory pathway, II) determine the corresponding preferred lateralization of sound in nuclei of the auditory pathway, and III) study the functional connectivity levels between these nuclei. We measured these three parameters in subjects without tinnitus and compared those to findings in subjects with unilateral tinnitus and near-normal hearing thresholds.

Parts of the data were presented previously [9], describing increased sound-evoked activity in the inferior colliculi (IC), but not in auditory cortex, of patients with unilateral tinnitus. Data of new subjects (four more controls, four more patients with unilateral tinnitus) were acquired and analyzed. In addition to sound-evoked activity, the current study focuses on the lateralization and connectivity patterns.

## Materials and Methods

### Subjects

Fourteen subjects with unilateral tinnitus were recruited at the University Medical Center Groningen, all without neurological and psychiatric history. Additionally, sixteen subjects without tinnitus were recruited. Hearing thresholds were obtained using standard pure-tone audiometry at the octave frequencies from 250 to 8000 Hz. All subjects were selected to have near-normal hearing. Compared to the previous study [9], four more controls and four more patients were included (one with right-sided tinnitus, three with left-sided tinnitus).

Subjects were selected to have a maximum averaged difference in hearing thresholds between the left ear and right ear of 10 dB in the range of 250–2000 Hz. A trained audiologist assessed all participants and the subjects with tinnitus were asked about various tinnitus characteristics such as etiology, tinnitus laterality, type, and severity. The following measures were determined in each tinnitus patient: (1) the frequency of a tone contralateral to the tinnitus ear, best matching the pitch of the tinnitus, (2) the level of a contralateral tone (in dB SL) at this frequency, best matching the tinnitus loudness, (3) the minimum masking level (MML) defined as the lowest level of an ipsilateral, narrowband noise centered at the tinnitus matching frequency, that fully masked the tinnitus. Eleven out of 14 subjects completed a Tinnitus Reaction Questionnaire, TRQ [17]. Finally, the handedness of each subject was determined using a translated version of the Edinburgh inventory [18]. General subject characteristics are summarized in Table 1 and a more detailed description of the tinnitus subjects can be found in Table 2. The study was approved by the local medical ethics committee (Medical Ethics Committee (METc) of the University Medical Center Groningen). All subjects were informed about the purpose of the study before giving written informed consent prior to testing.

**Table 1:** General subject characteristics.

	Controls	Left-sided tinnitus	Right-sided tinnitus
Characteristics	*(n = 16)*	*(n = 8)*	*(n = 6)*
Age (years)			
average	39.1	46.5	52.8
standard deviation	16.6	8.1	13.1
range	23–76	40–62	31–76
Gender			
male	8 (50%)	4 (50%)	4 (67%)
Tinnitus			
average pitch (kHz)	-	8.0	7.4
range (kHz)	-	0.8–14.0	3.0–11.0
average loudness (dB SL)	-	23	21
range(dB SL)	-	5–38	5–45
average MML (dB SL)	-	46	41
range (dB SL)	-	19–69	16–65
Handedness			
right handed	14 (88%)	6 (86%)	5 (83%)

**Table 2:** Tinnitus characteristics.

Sub	Age	Sex	Tinnitus laterality	Tinnitus duration (y)	Incident triggering tinnitus	Tinnitus quality	Tinnitus Pitch (kHz)	Tinnitus Loudness (dB SL)	MML (dB SL)	Severity	TRQ
10	55	m	right ear	2	unknown	pure-tone	3	5	16	mild	32
13	50	m	right ear	4	noise trauma	n/a	6	16	30	n/a	29
16	65	f	right ear	3	unknown	pure-tone	11	18	43	mild	33
18	67	f	right ear	5	unknown	ringing/pulse	6	20	n/a	mild	3
19	31	m	right ear	14	noise trauma	ringing	10	45	53	mild	25
29 (*)	49	m	right ear	1	blow on the ear	pure-tone	8	23	65	mild	32
Avg	52.8	-	-	4.8	-	-	7.4	21	41	-	24
7¶	49	m	left ear	6	car accident	pure-tone	8	35	70	mild	26
9	53	m	left ear	4	unknown	ringing	8	23	40	mild	9
11	62	f	left ear	6	flushing the ear	buzzing	8	15	48	severe	74
14	42	f	left ear	12	unknown	cricket	14	20	68	mild	61
17	37	f	left ear	9	unknown	hissing	8	25	43	severe	89
27 (*)	43	f	left ear	1	unknown	ringing	0,75	12	n/a	mild	n/a
28 (*)	40	m	left ear	0.5	unknown	pure-tone	8	13	19	severe	n/a
30 (*)	46	m	left ear	2	unknown	pure-tone	9	38	38	mild	n/a
Avg	46.5	-	-	5.1	-	-	8.0	23	46	-	52

n/a: not available; n: no; y: yes;

¶ one subject (subject #7) was excluded from analyses due to gross motion artefacts that occurred during the imaging sessions; MML: minimum masking level; TRQ: Tinnitus reaction Questionnaire.

(*) indicate new subjects with respect to Lanting et al., (2008) [9].

### Imaging paradigm

All imaging experiments were performed on a 3 T MRI system (Philips Intera, Philips Medical Systems, Best, The Netherlands) with an eight-channel phased-array head coil (SENSE head coil). A T1-weighted fast-field echo scan was acquired for anatomical orientation (TR 11.1 ms; TE 4.6 ms; flip-angle 15°; matrix 256×256×9; voxel-size 1.0×1.0×2.0 mm^3^). The functional imaging session included three 8-min runs, each consisting of a dynamic series of 51 identical 2200-ms single-shot T2*-sensitive echo planar imaging (EPI) volume acquisitions (TR 10 s; TE 22 ms; flip-angle 80°; matrix 128×128×41; voxel-size 1.0×1.0×2.0 mm^3^; interleaved slice order, no slice gap; SENSE reduction factor 2.7), and were acquired using a coronal orientation, aligned to the brainstem when viewed on a midsagittal cross-section. The influence of acoustic scanner noise was reduced by using a sparse sampling strategy [19], [20] in which auditory stimuli were presented during a 7.8-s gap of scanner silence between the end of each acquisition and the onset of the successive one. An additional 3D T1-weighted fast-field echo scan (TR 25 ms; TE 4.6 ms; flip-angle 30°; matrix 256×256×160; voxel-size 0.94×0.94×1.0 mm^3^) was acquired with the same orientation as the functional scans to serve as anatomical reference.

### Sound stimuli

Auditory stimuli were delivered by an MR compatible electrodynamic system (MR Confon GmbH) [21]. This system was driven by a PC setup equipped with a digital-to-analog converter (National Instruments 6052E, National Instruments Corporation, Austin, TX) controlled by Labview 6.1 (National Instruments Corporation, Austin, TX). The auditory stimuli consisted of temporally and spectrally modulated broadband ’dynamically rippled' noise [22]. The stimuli had a frequency range of 125−8000 Hz with a spectral modulation density of 1 cycle per octave, a temporal modulation frequency of 2 cycles per second and a modulation amplitude of 80%. The rippled noise stimuli were presented immediately when an MR acquisition started and ended 0.5 s before the next acquisition. All stimuli were 9.5 s in duration. Stimuli were presented at 40 or 70 dB (SPL) either at the left or the right ear. The stimuli were presented in a pseudo-randomized order. Each condition (four in total) was presented ten times per functional run. An additional ’silent' condition (i.e., no stimulus) was presented eleven times. Subjects were instructed to respond by left or right button presses with the right thumb whenever they perceived an audible stimulus in the left or right ear, respectively. This was done to monitor the subjects' attention to sound stimuli during acquisition.

### Data analysis

MR images were analyzed using Matlab 7.1 (R14) (The Mathworks Inc., Natick, MA) and SPM8 (Functional Imaging Laboratory, The Wellcome Department of Imaging Neuroscience, London, UK, http://www.fil.ion.ucl.ac.uk/spm/). The functional images were corrected for motion and spatially coregistered with the T1-weighted high-resolution anatomical image. The high-resolution anatomical image was segmented in grey matter, white matter and cerebrospinal fluid segments. The gray-matter segment of the anatomical image was normalized to a custom normalization template (for more details, see [9]) and the resulting transformation parameters were also applied to the functional data. The normalized functional data were spatially smoothed using an isotropic Gaussian kernel with a full width at half maximum of 4 mm, to improve signal-to-noise ratio characteristics while retaining the ability to discern small auditory structures (i.e., the brainstem nuclei). Functional images were interpolated to voxel dimensions of 2.0×2.0×2.0 mm^3^.

A general linear model (GLM) was set up for each subject to analyze the relative contribution of each stimulus condition to the measured response. The GLM included four covariates of interest, one for each condition, one constant factor to model the baseline signal or the signal during the silent condition and a linear term to correct for linear drift in the scanner signal. The GLM was applied to the data of all voxels and four contrast images were created, one for each condition (i.e., left 40 dB vs. baseline (L40), left 70 dB vs. baseline (L70), right 40 dB vs. baseline (R40) and right 70 dB vs. baseline (R70)). An omnibus F-test, including all four conditions, was assessed to detect the combined effect of all sound stimuli.

The four contrast images (per subject) were entered in a second-level random-effects analysis based on a flexible factorial design with factors for group (i.e., controls, subjects with tinnitus perceived on the left side and subjects with tinnitus perceived on the right side), subject, and stimulus condition.

In addition to the random-effects analysis, a non-parametric permutation test was performed to assess potential differences in the responses between the two patient groups. We used SnPM (http://www.sph.umich.edu/ni-stat/SnPM/) and permuted the labels of the two patient groups (i.e., right-sided tinnitus and left-sided tinnitus) and assessed whether the actual differences between groups were significant based on both the t-statistic and cluster size [23].

### Region of interest analysis

Following the voxel-wise analyses, we performed a region of interest (ROI) analysis, determining sound-evoked responses in 10 anatomical areas comprising (parts of) the auditory pathway and one area in the vermis of the cerebellum that was included based on previous findings [24]. The left and right primary auditory cortices were defined as the combination of the TE1.0, TE 1.1 and TE 1.2 areas defined by the SPM Anatomy toolbox [25]–[27]. For the left and right auditory association cortices (AAC) we used the left and right superior temporal gyrus as defined by Brodmann (BA 22) based on the AAL template in MRIcron (http://www.sph.sc.edu/comd/rorden/mricron/). Both of the ROIs of the primary and association cortices were normalized to match our anatomical template in order to have a corresponding image space. The left and right medial geniculate body of the thalamus (MGB), the left and right inferior colliculi (IC), the left and right cochlear nuclei (CN), and the ROI consisting of the vermis of the cerebellum were manually drawn based on an anatomical atlas [28], [29]. We used the xjView (http://www.alivelearn.net/xjview8) Matlab toolbox to select a relatively large volume around these nuclei, thus allowing for small differences between subjects that remain after normalization. Table 3 shows the size of each ROI, measured in voxels (of 2×2×2 mm^3^), and the location of their center of mass (given in MNI coordinates).

**Table 3:** Volume and center of mass of each ROI.

ROI	left hemisphere		right hemishpere	
	*voxels*	*location*	*voxels*	*location*
Auditory association cortex (AAC)	1339	(−58, −28,6)	1569	(60, −30,6)
Primary auditory cortex (PAC)	469	(−46, −16,4)	563	(48, −14,0)
Medial geniculate body (MGB)	53	(−16, −26, −8)	63	(16, −26, −8)
Inferior colliculus (IC)	29	(−6, −36, −12)	33	(4, −36, −12)
Cochlear nucleus (CN)	63	(−8, −37, −44)	52	(8, −38, −42)
Cerebellum vermis	287	(0, −54, −4)	-	-

The volume is measured in number of voxels (2×2×2 mm^3^) and the center of mass is given in MNI coordinates.

Based on the single-subject F-test (sound vs. baseline), the 10% most active voxels in each ROI were used (i.e., those exceeding the 90th percentile of the distribution of F-values).

A percentage signal change was calculated for each of these ROIs. First, the regression coefficients of the selected voxels within the region of interest were averaged for each condition separately. Next, these values were divided by the average baseline level of activity for the same voxels in order to get a percentage signal change.

To test for differences between the subject groups and potential interactions between the groups and the experimental conditions, repeated measures ANOVAs were performed for each ROI separately, using the percentages signal change obtained earlier. Two main factors were defined: (I) stimulus condition (L40, L70, R40, and R70) as repeated measure (within subject) and (II) subject group (controls, left-sided tinnitus and right-sided tinnitus). In addition, the interaction between the two main factors was assessed (group × stimulus). A Bonferroni correction for multiple comparisons (i.e., over the number of tested ROIs) was applied. To rule out any influence of potential confounding variables we performed regression analyses of the activation levels in each of the ROIs with hearing thresholds (pure tone average for left and right ear separately), and age.

### Response lateralization

Since monaural stimuli were used, it was possible to determine the preferred stimulus lateralization. From the ROI analysis we obtained for each subject the mean response for each condition. The mean responses to left (L) and right ear (R) stimuli were calculated by averaging the response to the 40 and 70 dB (SPL) stimuli at each ear. A lateralization index (LI) was obtained for each of the regions of interest separately, defined as *LI* = *L–R*/(|*L*|+|*R*|), with possible outcomes ranging from –1 to +1 for unilateral positive responses to right and left ear stimulation, respectively.

### Connectivity analysis

For our connectivity analysis, we used the Pearson correlation coefficient [30], [31]. Our model consisted of ten auditory regions: the left and right CN, IC, MGB, PAC and AAC. In addition, the vermis of the cerebellum was included as an eleventh ROI. The mean signal of the 10% most active voxels within each ROI (i.e., those exceeding the 90th percentile of the distribution of F-values) was calculated for each point in time (i.e., for each scan). The obtained fMRI time courses of these ROIs were transformed to zero mean and unit variance for each subject. These arrays were concatenated over subjects resulting in a matrix containing 11 time courses of 2448 elements (16 subjects×153 time points) for the control group and a matrix containing 11 time courses of 1989 elements (13 subjects×153 time points) for the patient group. For each group, the covariance matrix **Σ** was calculated, which contains the Pearson cross-correlation for all possible ROI pairs, respectively.

To assess whether observed differences between the groups were significant, the Jensen-Bregman LogDet Divergence (JBLD), a dissimilarity measure for differences between covariance matrices, was used [32]. First, the observed JBLD (or dissimilarity) value was calculated using the two groups’ covariance matrices **Σ**. Next, we randomly permuted the assignment of subjects to the two groups (retaining the original group sizes), obtained new time courses and the resulting covariance matrices, and calculated the JBLD value for each of the permutations. This was repeated 50000 times and a reference distribution of similarity values was obtained. To assess whether the observed difference exceeded the significance level of *p* = 0.05, we calculated the proportion *p* of sampled permutations where the absolute difference was greater than, or equal to, the observed difference.

Second, we calculated the observed differences in correlation coefficients between the subject groups for the connection between each pair of ROIs separately. Similar to the permutation testing on the JBLD dissimilarity value, both the observed correlations and the reference distributions were obtained using the same set of permutations as before. Finally, the observed values were compared against the reference distributions and a *p-*value was obtained for each of the ROIs.

Potential confounding variables, such as age, average hearing thresholds, and average left-right differences in hearing thresholds were accounted for by analyzing the relation (or correlation) of the JBLD dissimilarity value with each of the variables for each of the permutations. The assumption here is that if any of the variables has a confounding effect on the JBLD dissimilarity values, these values would be correlated with the confounding variables. Conversely, it would be expected that in a subset of permutations with the same degree of e.g., hearing loss, its (confounding) effect on the dissimilarity value would be accounted for.

## Results

### Audiometry and tinnitus assessment

**Figure 1:**
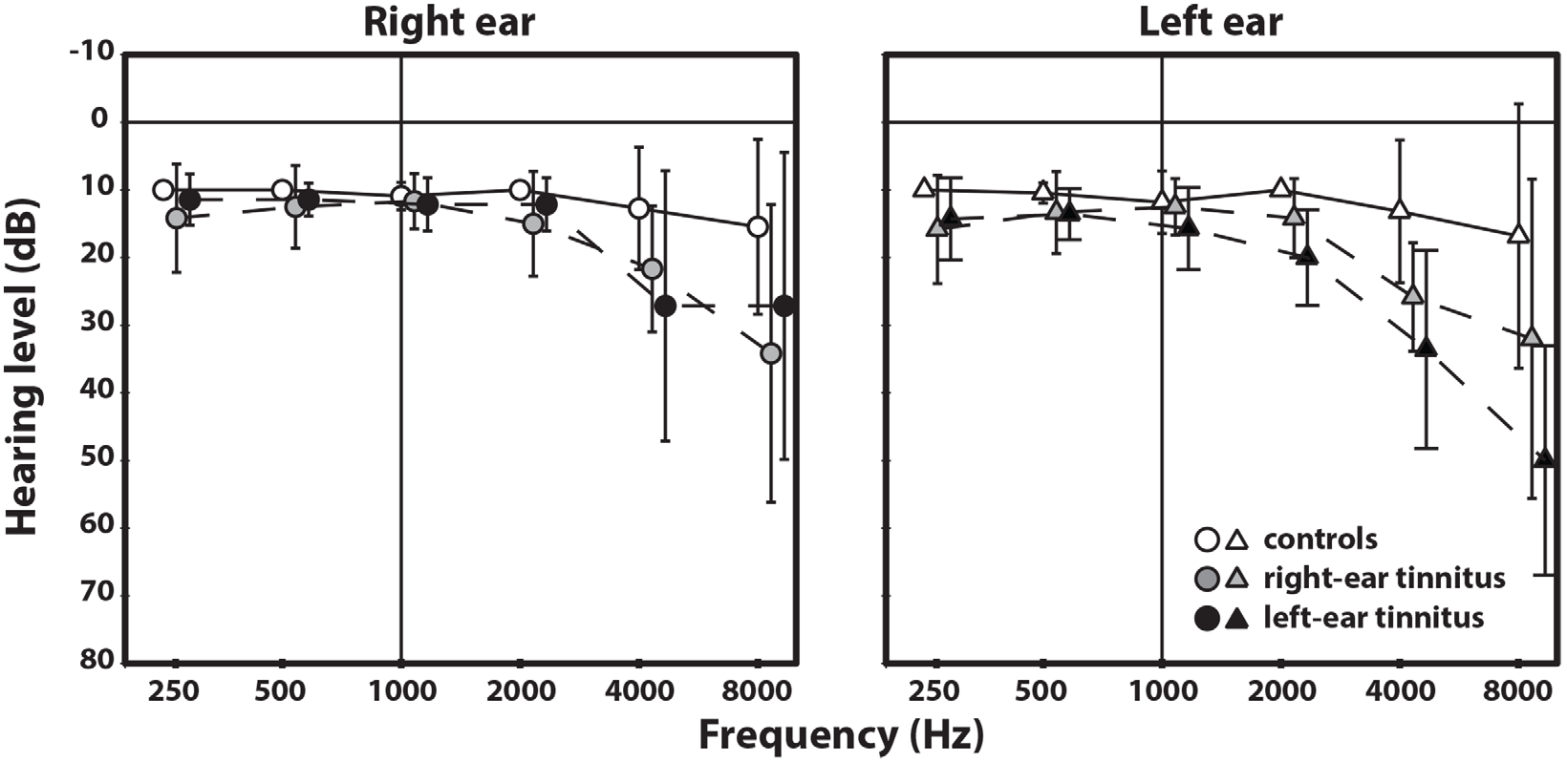
Mean pure-tone hearing thresholds for the right and the left ear for the three subject groups. The solid line represents the hearing thresholds of the control group and the two dashed lines represent the hearing thresholds of the two groups with unilateral tinnitus. The error bars indicate the standard deviation around the mean.

The mean audiogram of each group is displayed in Fig. 1. There were no significant differences between the average thresholds of the groups for the frequency range 0.25–2 kHz. At 4 and 8 kHz, the tinnitus subjects showed thresholds that were significantly elevated (*p*<0.01) relative to those of the controls whereas there was no significant difference (*p*>0.1) between the two groups of subjects with tinnitus. These two groups of subjects with tinnitus were reasonably well matched (see Tables 1 and 2) concerning the average values of the duration of the tinnitus (4.8 and 5.1 years) tinnitus pitch (7.4 and 7.9 kHz), the average loudness of the tinnitus (21 and 23 dB SL) and the average MML (41 and 46 dB SL). Thirteen of the 14 patients were asked to categorize the severity of their tinnitus as mild, moderate, or severe. Ten patients considered their tinnitus as mild whereas the other three reported their tinnitus as severe. This closely corresponded to the obtained TRQ scores, where available (severe: TRQ scores of 74 and 89; mild: TRQ scores between 9 and 61). For further details see Table 2.

### Sound evoked activation

**Figure 2:**
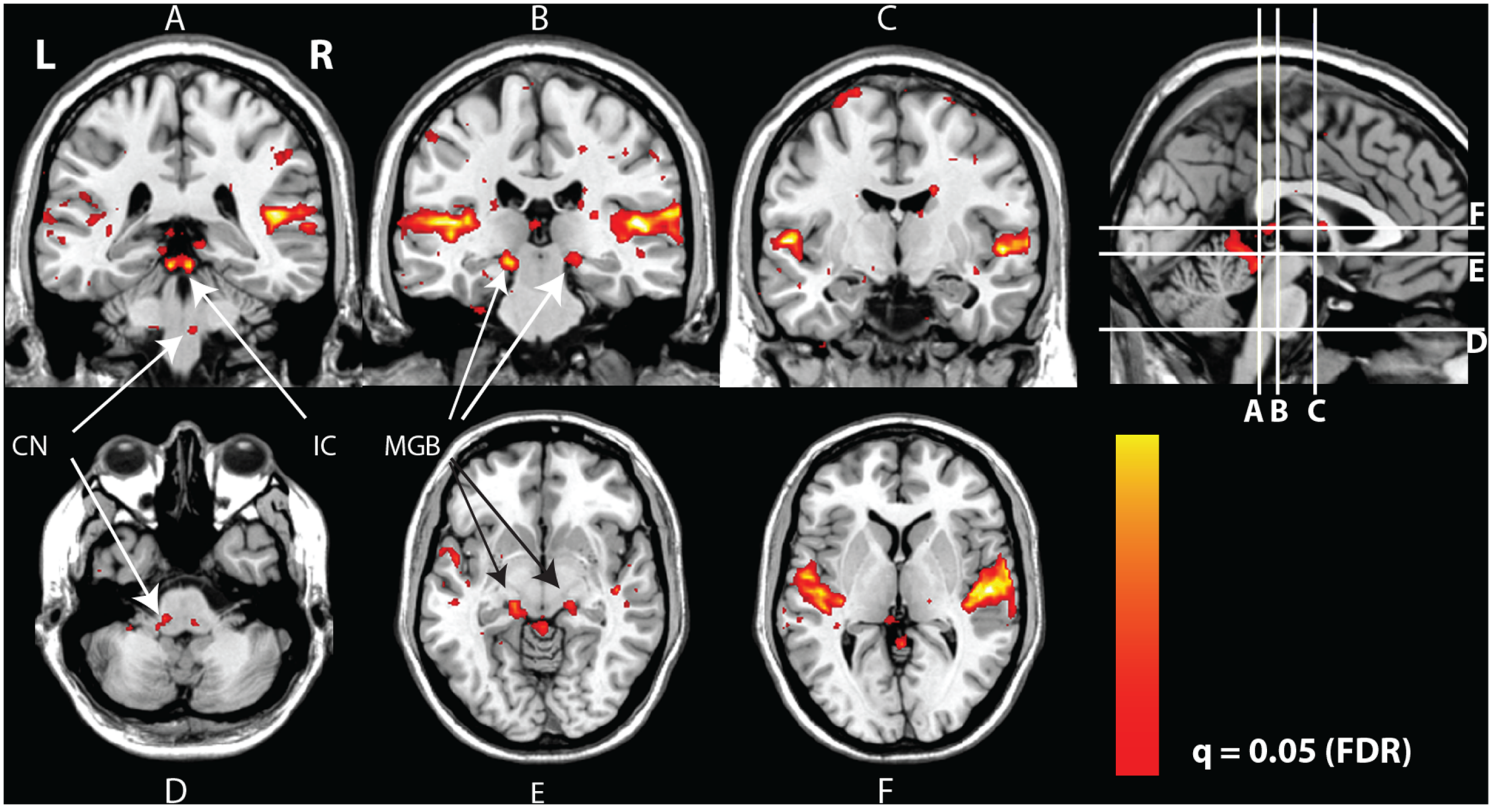
Sound-evoked responses. Coronal and transversal cross-sections of the human brain in grey-scale with a red-yellow color-coded overlay showing significant responses to sound. The colored areas show a significant response to sound stimuli (omnibus F-test, *F*>8.34, *q*<0.05 FDR, pooled over all subjects). Evident from this figure is the auditory pathway, showing the cochlear nuclei (CN; panel A and D), the inferior colliculi (IC; panel A and E), the medial geniculate bodies (MGB; panel B and E) and the auditory cortices (panels A–C and F).

**Figure 3:**
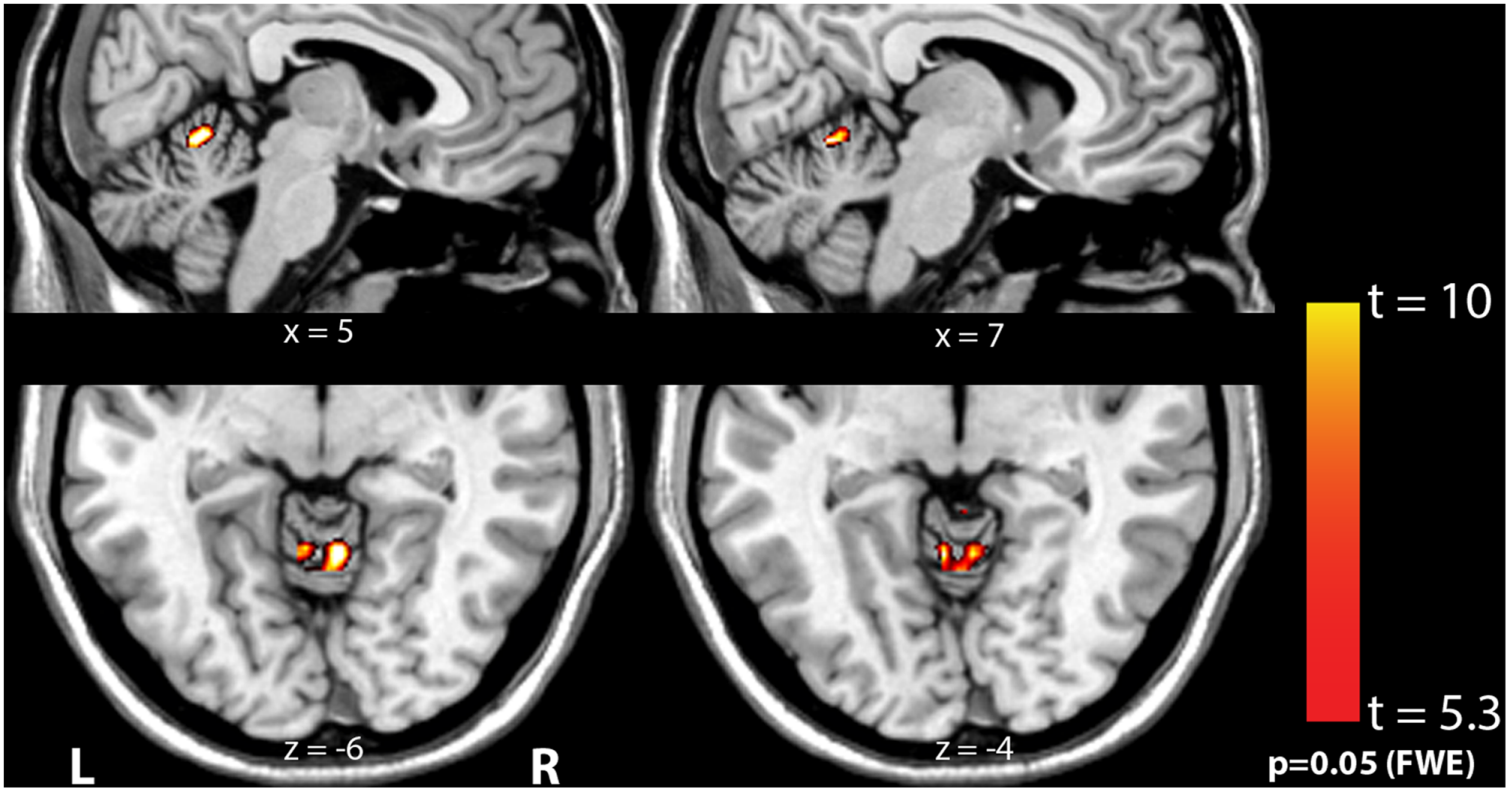
Coronal and sagittal cross-sections of the human brain in grey-scale with a red-yellow color-coded overlay showing voxels in the vermis of the cerebellum that show a significantly larger response to sound in patients compared to controls (*t*>5.34, *p*<0.05 FWE).

The group-level significance of the sound-evoked hemodynamic responses is displayed in Fig. 2 (*n* = 29; one subject (subject 7) in the group with right-sided tinnitus was excluded from further analyses due to motion artifacts). It clearly shows significant sound-evoked responses in the left and right CN, IC, MGB, and the bilateral auditory cortices (see table 4 for the location and F-value of the responses). When contrasting the whole patient group against the controls, no significant differences were observed, with the exception of the vermis of the cerebellum as shown in Fig. 3.

**Table 4:** Location of the maxima as in figure 2, one for each ROI (MNI coordinates) and their F-values (auditory ROIs) or t-value (vermis of cerebellum).

ROI	left hemisphere		right hemishpere	
	*location*	*statistical value (F)*	*location*	*statistical value (F)*
Auditory associationcortex (AAC)	(−44, −28,10)	19.3	(54,−18,4)	19.0
Primary auditorycortex (PAC)	(−36, −22,6)	16.1	(40, −22,6)	20.4
Medial geniculatebody (MGB)	(−14, −26, −6)	4.5	(16, −26, −6)	11.0
Inferior colliculus(IC)	(−6, −34, −12)	13.7	(2, −36, −10)	13.6
Cochlear nucleus(CN)	(−10, −36, −44)	4.0	(5, −34, −44)	4.0
Cerebellum vermis	(−2, −52,0)	14.5 (*t-value*)	-	-

The dissimilarity between the two patient groups was investigated by performing a non-parametric permutation test based on both the t-statistic and cluster size. Neither of these two measures showed any significant differences (*p* = 0.05 FWE) between the subject groups, indicating that responses were neither different in strength nor in extent. Because the lateralization of the tinnitus did not influence the strength or location of sound-evoked activation, we decided to pool the data from the two patient groups in a number of the analyses that followed.

### Region of interest analysis

**Figure 4:**
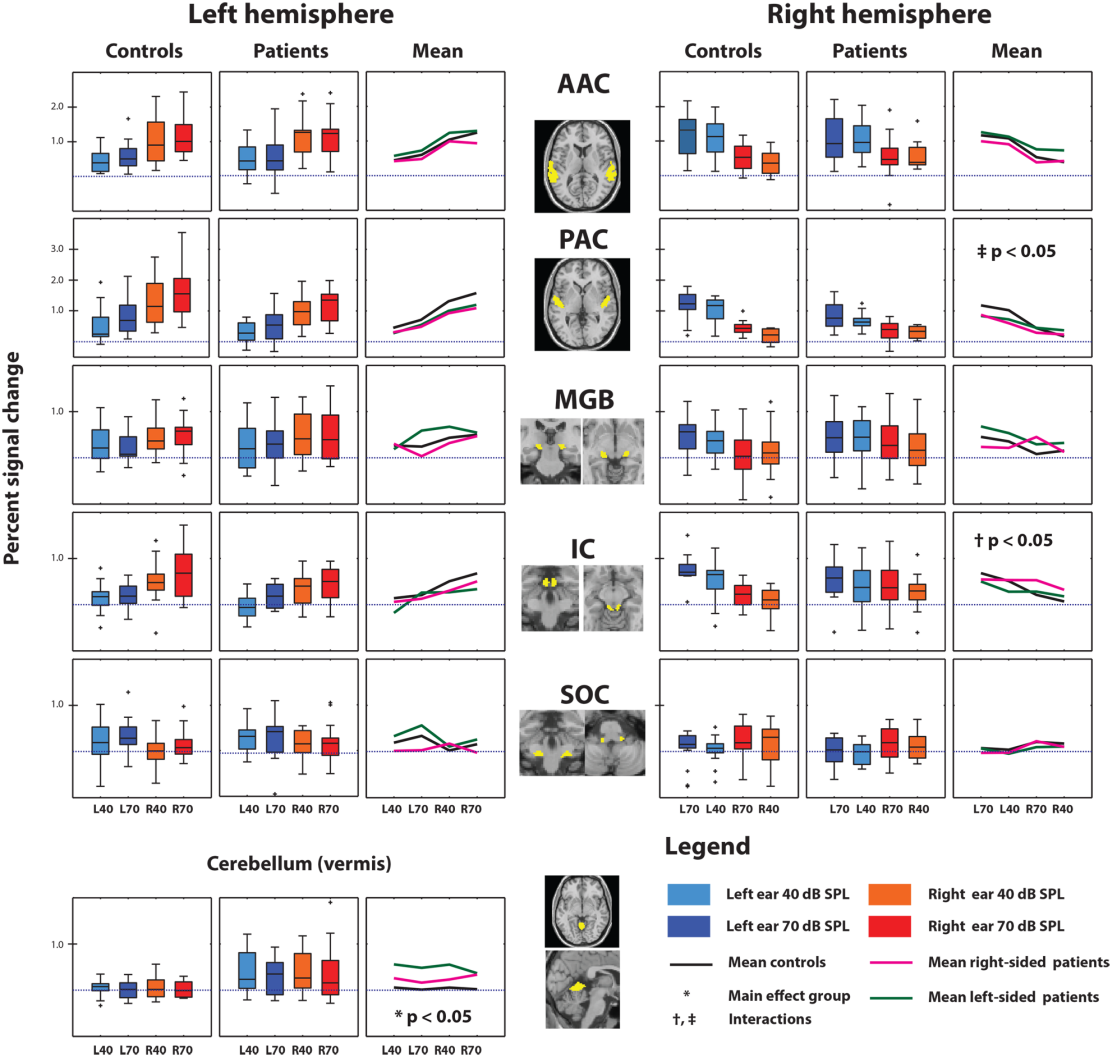
Region of interest analysis. The percentage signal changes measured in each ROI of the left and right hemisphere (AAC, PAC, MGB, IC and CN) and the vermis of the cerebellum for both subject groups. The location of each ROI is indicated in yellow on cross-sections of the brain. The responses to the four experimental conditions are shown as box plots for each group separately. For each group, the mean per condition is visualized in the line plot next to the box plots.

We performed ROI analyses, averaging the 10% most active voxels within each ROI, using ten ROIs in the auditory pathway and the vermis of the cerebellum. The box plots in figure 4 show the responses to the four experimental conditions, L40, L70, R40 and R70, for controls and subjects with tinnitus for the various ROIs. In addition, it shows the mean value per condition for each subject group. For the size and location of each of the ROIs please refer to Table 3.

With the exception of the CN, activation in the auditory pathway is strongest in response to the contralateral ear. For the CN we observe a weak ipsilateral preference. In addition, with the exception of the MGB, there is a clear sound intensity dependency, i.e., the 70 dB (SPL) stimuli yielded a larger response than the 40 dB (SPL) stimuli. The only ROI that showed a significant difference between controls and patients with tinnitus was the vermis of the cerebellum (*p*<0.05, corrected for multiple comparisons). For all conditions, both patient groups clearly showed a larger response in the vermis of the cerebellum than controls (see Fig. 4). Finally, the right PAC and right IC showed a significant interaction of group × condition (*p*<0.05, corrected for multiple comparisons). Patients, on average, showed a smaller difference between the ipsilateral (right-ear) stimuli and the contralateral (left-ear) stimuli than the controls in these ROIs (see Fig. 4).

To rule out any influence of hearing loss in the left and right ear, age, and the TRQ value, we performed regression analyses on the percentage signal change with each of these factors as explanatory variables. These showed that these potential confounds could not account for the differences between controls and tinnitus patients (*p*>0.1 in all ROIs).

### Response lateralization

**Figure 5:**
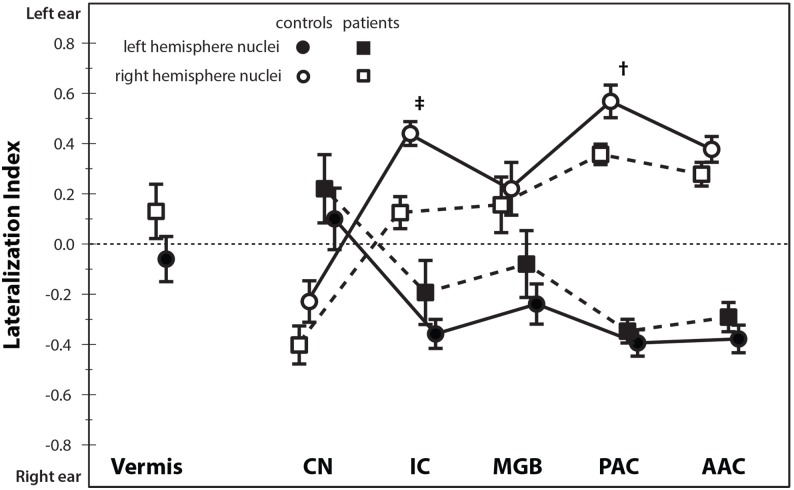
Sound lateralization in the auditory pathway. The lateralization indices for the left hemisphere nuclei (filled symbols) and the right hemisphere nuclei (open symbols) of the auditory pathway (AAC, PAC, MGB, IC and CN) and the cerebellum. A lateralization index of +1 indicates a response to left-ear stimuli only, whereas a value of −1 indicates a response to right-ear stimuli only. The error bars indicate the standard error of the mean. The symbols indicate the two nuclei (†: PAC and ‡: IC) where the difference in lateralization index is significantly different between the two patients and controls.

Fig. 5 shows the preferred stimulus lateralization index for each nucleus for the controls and for the pooled patient groups. Post-hoc analysis using permutation testing revealed that the two patient groups did not differ significantly (*p*>0.1). The ipsilateral lateralization of the CN and the contralateral lateralization of the IC, MGB, PAC, and AAC are clearly visible. The PAC showed the strongest contralateral lateralization, followed by the AAC, and the IC each with a contralateral lateralization, whereas the MGB shows a weaker lateralization. The vermis of the cerebellum, in contrast, did not show a clear lateralization (which can also be observed from Fig. 4). Significant group differences were observed in the right PAC (*p*<0.05) and right IC (*p*<0.001). In these nuclei, the lateralization index was significantly lower in subjects with tinnitus than in controls. Overall, the lateralization index was closer to zero in patients than in controls (repeated measures ANOVA, p<0.01) regardless of the lateralization of the tinnitus. Since differences between left- and right ear hearing thresholds might have an influence on the observed lateralization, we calculated the for each subject the root-mean-square (RMS) difference in hearing thresholds, taking into account differences between the left- and right ear thresholds across all frequencies. This analysis showed that, although the variability of this left-right ear asymmetry is smaller in the control group than in the patient group (5.9 and 7.4 dB, respectively), they do not differ significantly (p = 0.34, based on permutation testing, randomly assigning the group label and calculating the difference between the groups).

### Connectivity analysis

**Figure 6:**
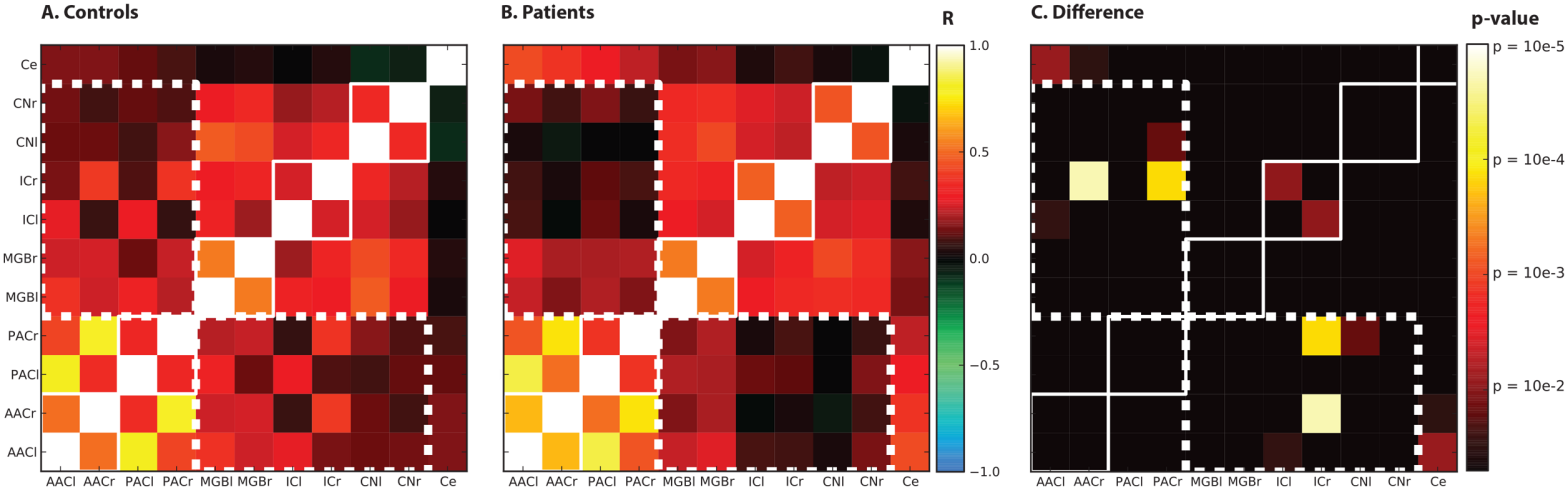
Connectivity patterns. Observed functional connectivity patterns in controls (**panel A.** **Controls**) and subjects with tinnitus (**panel B - Patients**). Pearson cross-correlation coefficients were calculated and color-coded based on the value of the coefficient. **Panel C. Differences** shows the differences in connectivity measures between subject groups for the different ROIs. Significance maps are associated with the observed difference between controls and patients for the Pearson correlation coefficients for each connection. The solid white lines represent homologue auditory nuclei at each level and the white dotted lines indicate the set of connections where on average the connectivity between subcortical and cortical areas is decreased in patients.

We calculated the Pearson correlation coefficients (see Fig. 6) between all nuclei that were included in the ROI analysis. The strongest Pearson correlations in the control group were observed between the ipsilateral PAC and AAC (0.78, 0.79 for left and right, respectively), and the left and right homologous nuclei at each level of the auditory pathway, varying between 0.25 (IC) and 0.53 (MGB). The ipsilateral connections between the IC and PAC and IC and AAC were also relatively strong (range: 0.27–0.40), whereas the contralateral connections were less strong (range: 0.07–0.14). The connections of both the bilateral IC and CN with the bilateral MGB nuclei were also notable (0.18–0.47), but without a clear lateralization.

The pattern of connectivity of the patient group was similar to that in controls in the sense that the strongest Pearson correlations were observed between left and right homologous nuclei at each level of the auditory pathway, varying between 0.38 (PAC) and 0.66 (AAC), and between the ipsilateral PAC and AAC (0.85, 0.74 for left and right, respectively). The connectivity pattern in the patient group, however, was distinctly dissimilar in two ways. First, there was a strong connection of the cerebellum with the PAC and AAC (range: 0.22–0.43) in the patients, compared to lower values in the controls (0.08–0.15). Second, the pattern of the correlation coefficients between the cortical areas (PAC and AAC) on the one hand, and the subcortical areas (MGB, IC and CN) on the other hand, was qualitatively different than that in the controls (see the white dotted outline in figure 6). Expressed as an average correlation coefficient, this value is lower in patients (0.11) than in controls (0.21). The same holds for the average correlation between the thalamus and the cortical areas: the patients show a lower value than in control (resp. 0.20 and 0.31).

To assess the statistical significance of these differences, they were also expressed in the Jensen-Bregman LogDet Divergence (JBLD). Compared to all the possible permutations, the actual dissimilarity was significantly higher (*p* = 0.002). The covariance matrices thus detectably differed between the groups. Permutation testing was also used to assess which of the connections was driving this difference, or in other words, which of the individual connections was significantly different between the groups. This is visualized in the rightmost panel of Fig. 6 as a significance map for the differences between the controls and patients for the Pearson correlation. The most prominent differences in Pearson correlation between the controls and patients related to the connections between the left and right IC, between the right IC and right PAC, and between the right IC and the right AAC.

To assess the influence of potential confounding variables, such as differences in hearing loss and age, and differences between left and right ear hearing thresholds, we analyzed the correlation of these variables with the JBLD dissimilarity values for all permutations. For example, if the dissimilarity was driven by differences in age between the subject groups, one would expect the JBLD dissimilarity value to be correlated with the group age-difference. In other words, plotting the dissimilarity value versus age for all possible permutations of age-difference between the groups would show a correlation between the two variables.

However, age only weakly correlated (r = −0.05) with the dissimilarity values (Fig. S1); Moreover, when looking in a range of 2 years around the actual measured difference between the groups, the permutation analysis showed that the measured dissimilarity is significantly larger than expected (p = 0.002 based on n = 3320 permutations within the selected age-bin), indicating that age cannot account for the dissimilarity between groups.

The same applies to differences in hearing loss, taken as the difference in average hearing thresholds at 4 and 8 kHz between the groups, where a small correlation coefficient of r = −0.02 was found between the dissimilarity index and hearing loss (Fig. S2). As with age, a significantly dissimilarity was found (p = 0.006 based on n = 2336 permutation within the selected bin; actual HL difference ±5 dB), indicating that differences of the hearing thresholds between the groups are not likely to have had a strong influence on the dissimilarity index.

Finally, we assessed whether differences in hearing thresholds between the left and right ear between the groups were of influence. This seems not the case as there is no strong correlation with the left-right differences in thresholds and the dissimilarity index (r = 0.03), and the dissimilarity is significantly larger than expected in a subset of the permutations (Fig. S3).

## Discussion

Tinnitus is an auditory phenomenon that in many patients is related to peripheral hearing loss. Yet, its pathogenesis is believed to be based on mechanisms in the central auditory system [7], [33]–[35]. If auditory processing by the brain is indeed different between subjects with and without tinnitus, this may result in differences in the way the brain responds to sound. Hence, we measured the response to sound in subjects with unilateral tinnitus en controls without tinnitus, all having normal or near-normal hearing.

Our findings are summarized as follows: (1) the amplitude of the sound-evoked brain responses was similar in all auditory brain areas of subjects with vs. without tinnitus; (2) tinnitus subjects displayed an enlarged response to sound in the cerebellum; (3) the lateralization of the response was less pronounced in tinnitus subjects than in controls; (4) there was no correspondence between lateralization of the sound-evoked responses and the lateralization of the tinnitus percept; (5) connectivity measures differed between tinnitus subjects and controls and showed decreased subcortical-cortical connectivity patterns in patients compared to controls.

### Sound-evoked responses in the auditory pathway

Changes in the sound-evoked responses in the auditory pathway have been previously linked to tinnitus, most notably with changes in the response characteristics of the inferior colliculus (IC) in subjects with tinnitus compared to controls [9]–[11], [36]. As mentioned, parts of the data of the current paper have been described earlier [9]. The current study added four controls and four tinnitus patients to the subject groups. In contrast to the earlier analysis [9], the current analysis did not show increased sound-evoked activity in the tinnitus patients (see Fig. 4).

In order to explain the apparent discrepancies, we highlight the effect of adding new subjects as well as the effect of methodological differences. Performing the old analysis on the full subject group included in the current work (see Fig. S4.) confirms previous results: sound-evoked responses as measured with this method are increased in the IC of patients with tinnitus compared to controls [9]. However, for our current analysis the voxel selection-criterion changed in two ways with respect to earlier [9]. Previously, IC ROIs were drawn manually for each subject. In contrast, we now used an objective method, in which a standard anatomical atlas was used to determine the location of the IC. A probability map, showing the overlap between ROIs across subject shows that the old and new ROI overlap nicely, but that the individual (old) ROIs are bigger in size (see the inset in Fig. S4).

The second difference is that previously the 10% of the voxels with the highest t-values were selected for each stimulus condition separately. Consequently, the voxels considered were not necessarily identical across conditions. However, the connectivity analysis employed in the current study requires the selection of a fixed set of voxels across conditions. Therefore, an omnibus F-test was used, selecting a fixed set of voxels for all stimulus conditions. As is evident in Fig. 4 and Fig. S4, with the new voxel selection criterion, there are no differences between tinnitus subjects and controls in the response amplitudes of the inferior colliculus.

It is of interest that the method used by Lanting et al. (2008) does show a difference between tinnitus subjects and controls, while the current method does not. At present we have no clear interpretation of this effect, but it warrants further investigation in future research.

Apart from these differences there is a more fundamental issue with increased sound-evoked responses and tinnitus. That is because it has been shown that subjects without clinical hyperacusis but with decreased loudness discomfort levels (LDLs) show increased sound-evoked responses in the IC [11]. Increased sound-evoked responses thus seem a proxy for hyperacusis but not necessarily for tinnitus, at least at the level of the IC. Unfortunately, since neither previously [9] nor in this work we have measured LDLs, it may be the case that some of the patients that show increased sound-evoked responses do so because of hyperacusis rather than their tinnitus.

The results presented here show that we did not find increased sound-evoked responses in patients with tinnitus but that it depends –at least partly- on the ROI definition, The analysis also revealed a clear level dependency in the cortex, thalamus and midbrain (Fig. 4). The response in each of the auditory nuclei increased with increasing level, which is in agreement with earlier findings [9], [37]–[40]. Moreover, the ROI analysis shows that the AAC, PAC, IC, and -to a lesser degree- the MGB show response lateralization; activation occurred most strongly in response to stimulation of the contralateral ear. The CN shows strongest activation in response to stimulation of the ipsilateral ear, as would be expected.

### The cerebellum and tinnitus

The only brain region where the response to sound significantly differed between subjects with tinnitus and controls was the vermis of the cerebellum (see figure 3) Although the role of the cerebellum in auditory processing is largely unknown, there are a few studies that show a cerebellar association with sound processing. For example, connections between the CN and the cerebellum indicate that the cerebellum receives auditory input [41]–[43]. In addition, the vermis is thought to play a specific role in binaural processing, where auditory cues are used to control, for example, neck muscles to move the head towards a sound source [44], [45]. Finally, in humans and other animals alike, lesions in the medial part of the cerebellum are associated with a lack of long-term habituation of the acoustic startle response [46]–[48].

There is also some evidence for the role of the cerebellum in tinnitus. Brozoski and colleagues found that, in addition to elevated levels of activity in the auditory brainstem, there was also increased activity in the paraflocculus of cerebellum of rats with behavioral tinnitus [49]. In subjects with gaze-evoked tinnitus, the vermis seems to be more activated compared to controls [50]. Finally, as previously reported, a cerebellar response to sound was found in normal hearing controls but not in tinnitus subjects that were able to modulate their tinnitus by jaw protrusion [24]. On the whole, the evidence suggests a role of the cerebellum in tinnitus. The exact nature of this role, however, remains unclear at the moment.

### Reduced lateralization and tinnitus

The lateralization index (see Fig. 5) is a quantity that summarizes the relative response of a brain area (e.g. a ROI) to stimulation of the right and left ear, respectively. The index was significantly lower in the right primary auditory cortex and the right inferior colliculus in patients than in controls, indicating a less pronounced preference for responding to the contralateral ear. Moreover, the lateralization was far less pronounced in subjects with tinnitus than in controls. The reduced lateralization, at least at the level of the IC, in combination with the significant interaction (group x condition; see Fig. 4), is in line with previous work that showed no clear response lateralization in the IC in patients with unilateral tinnitus [9]. Importantly, analyses showed that this reduced lateralization is not due to inherent differences between left- and right ear hearing-thresholds.

This decreased lateralization might relate to a diminished efficiency in the inhibitory ipsilateral input to the IC. Where the contralateral pathway receives mainly excitatory input, the ipsilateral pathway receives both inhibitory and excitatory input [51]. A reduction in the inhibitory pathway could thus lead to a more equal input from both ears via normal contralateral excitatory input and, through normal excitatory and reduced inhibitory input, relatively more excitatory input from the ipsilateral ear. This, in turn would lead to a decrease in the lateralization index.

Remarkably, the preferred stimulus lateralization indices show no relation to the lateralization of the tinnitus both in this work and previous papers [9], [10]. Normally, sound from one ear is predominantly represented in the contralateral hemisphere [52]. Thus, tinnitus in e.g. the left ear would be expected to correspond to aberrant neural activity in the right cortex. Yet, we find a bilateral diminished lateralization. Apparently there is no clear relation between the lateralization to sound in subjects with tinnitus with the lateralization of the tinnitus. Note, however, that the absence of a clear difference in lateralization between the two patient groups may be due to the relative small sample-size of patients with eight and six patient for respectively the left- and right-sided tinnitus patient group. This point makes that the conclusion may not be easy to generalize and that there may be more subtle differences in lateralization.

### Changes in connectivity patterns

Finally, we studied the connectivity patterns between nuclei of the auditory pathway in a similar fashion as was previously done in subjects with unilateral hearing loss [52] and recently in subjects with mild to severe hearing loss and tinnitus [16]. In functional MR imaging, functional connectivity measures express the degree of similarity of the measured signals in time in various areas of the brain. Activity that co-varies suggests that the neural processes underlying this activity are related. Simple (Pearson) cross-correlations between ROI responses were computed, a measure that is usually referred to as functional connectivity [31]. Yet, the connectivity patterns reported here do not necessarily match direct anatomical connectivity patterns obtained using e.g. anterograde and retrograde labeling techniques in animals [53], or non-invasive diffusion tensor imaging in humans [54]. The functional connection between two nuclei may be related to shared input that both receive, or mediated via a third nucleus using an indirect path. The connection between e.g. the left and right MGB is an example of a connection that is likely to involve shared input from the IC, rather than an actual direct connection. Functional connectivity therefore has its limitations in terms of anatomical connections and the direction of information from one area to another.

Thus, two studies in patient populations that differ in their degree of hearing loss, respectively mild to moderate sensorineural hearing loss [16] and no hearing loss to mild high-frequency hearing loss (this study), show consistent differences in the connectivity patterns of patients compared to controls. The connectivity pattern was found to differ significantly between groups. More specifically, we showed a decreased (average) correlation between the cortical and subcortical clusters in tinnitus patients compared to controls. Importantly, these differences do not seem related with confounding variables such as age and hearing loss (figures S1–S3). Since the connection between both clusters comprises the thalamus, conveying information from the brainstem to the auditory cortex, it is possible that the difference in functional connectivity is related to a thalamic dysfunction.

According to one of the models of chronic tinnitus, the nucleus accumbens and associated paralimbic areas in the ventromedial prefrontal cortex (vmPFC) play an important role in long-term habituation to continuous unpleasant sounds [12]. Sound-evoked neural activity is normally relayed from the auditory periphery via the brainstem and thalamus to the auditory cortex for conscious perception. The same signal is also directed via the amygdala to the nucleus accumbens for evaluation of the sound’s emotional content [55]. From the nucleus accumbens, projections feed back to the thalamic reticular nucleus (TRN), which in turn selectively inhibits the sections of the thalamus corresponding to the irrelevant sound frequencies. This gain-control mechanism leads to filtering of unwanted sounds, which then do not reach conscious perception in the auditory cortex. As long as this feedback system is intact, the tinnitus signal is filtered out. If, however, parts of the feedback system have become compromised, such as lesions that translate in decreased gray matter volumes in (parts of) the vmPFC [55]–[57], the abnormal sound may be passed on to the cortex causing the conscious perception of tinnitus.

In a different model, tinnitus seems to be related to changes in thalamocortical rhythms that naturally occur in the brain [14]. As a consequence of hearing loss and reduced input to the thalamus, normal rhythms of the thalamocortical loop change to increased large-scale, slow-rate oscillatory coherent theta (4–8 Hz) activity, in turn reducing lateral inhibition and disinhibiting more high-frequent gamma (30–70 Hz) oscillations [15]. Tinnitus-related increases in the low-frequency delta-band (0.5–4 Hz) and decreases in the alpha-band (8–13 Hz) was previously shown [58].

For both models, it could be argued that tinnitus leads to reduced connectivity between thalamus and cortex, or even brainstem and cortex. If the thalamus is disinhibited (by e.g. reduced inhibitory input from the TRN) more excitatory activity reaches the cortex [12], ultimately leading to tinnitus. Due to this increased cortical (tinnitus-related) activity, there is little room for activity relating to other (non-tinnitus) sound stimuli, which may lead to a reduction in connectivity patterns.

Decreased connectivity also applies to the model by Llinas et al., (1999). Here deafferentation leads to reduced thalamic activity, like for example in patients with gaze-evoked tinnitus [59]. The decrease of thalamic activity may affect the normal flow of auditory processing from brainstem to cortex, possibly resulting in reduced connectivity as observed in this study. Thus the data observed show abnormal patterns of connectivity, compatible with both models, although the precise nature of the changes, and the exact role of the thalamus remain unknown.

In conclusion, the connectivity and lateralization results show that tinnitus involves the interplay between multiple brain regions, both along and beyond the classical auditory pathway. Although the conscious perception of tinnitus is ultimately based on patterns of neural activity in the auditory cortex, this work indicates that tinnitus seems related to abnormal connectivity patterns between subcortical nuclei and cortical brain areas.

## Supporting Information

Figure S1 **Dissimilarity as a function of the age-difference between controls and patients.** Each of the points corresponds to one permutation. For each of the 50000 permutations the dissimilarity index and difference in age between the groups were determined. Their marginal distributions are displayed on top or at the right side, respectively. The red lines indicate the actual dissimilarity index (horizontal red line) and the actual difference in age (vertical red line). The blue lines indicate the 95-percentile range of the distributions.
(TIF)

Figure S2 **Dissimilarity as a function of the difference in hearing-level (HL) between controls and patients.** For each of the 50000 permutations the dissimilarity index and difference in HL between the groups were determined. Their marginal distributions are displayed on top or at the right side, respectively. The red lines indicate the actual dissimilarity index (horizontal red line) and the actual difference in age (vertical red line). The blue lines indicate the 95-percentile range of the distributions.
(TIF)

Figure S3 **Dissimilarity as a function of the difference in left-right hearing-levels between controls and patients.** For each of the 50000 permutations the dissimilarity index and difference in left-right hearing-levels between the groups were determined. Their marginal distributions are displayed on top or at the right side, respectively. The red lines indicate the actual dissimilarity index (horizontal red line) and the actual difference in age (vertical red line). The blue lines indicate the 95-percentile range of the distributions.
(TIF)

Figure S4 **Sound-evoked responses in the left IC in controls (panel A), subjects with right-sided tinnitus (panel B) and subjects with left-sided tinnitus.** The panels show four box plots per condition (left 40 dB, left 70 dB, right 40 dB, right 70 dB). The dark red bars show the average responses determined over the 10%most active voxels according to a (condition-wise) t-test in a manually drawn ROI. These results are identical to those reported by Lanting et al, (2008). The orange box plots represent results from identical analyses performed on the same subjects combined with the subjects that were added for this study (four controls, one patient with right-sided tinnitus and three with left-sided tinnitus; indicated with (*) in table 2). The dark blue box plots represent the analysis of the complete data-set with the identical t-test procedure again selecting the 10% most active voxel but using a ROI definition that was based on an anatomical template (MNI) and identical for each subject. The light blue box plots represent the results when an F-test (including all conditions) was used instead of the (condition-wise) t-test. The ROI selection has arguably the biggest effect on the size of the sound-evoked responses, especially in the patients groups. This effect is less dramatic in the controls. The insets (D–F) show the location and extent of the left IC ROIs. It shows a probability map, indicating the amount of overlap between subjects’ ROIs as used in the Lanting et al., 2008 study, thresholded at 80% overlap between all subjects in red-yellow colours. In blue is shown the template ROI as defined on the MNI template.
(TIF)
