# Supplementary figures and images for: Unilateral Tinnitus: Changes in Connectivity and Response Lateralization Measured with fMRI
Source: PLoS One. 2014 Oct 20;9(10):e110704. doi: 10.1371/journal.pone.0110704 (PMC4203817; doi:10.1371/journal.pone.0110704)

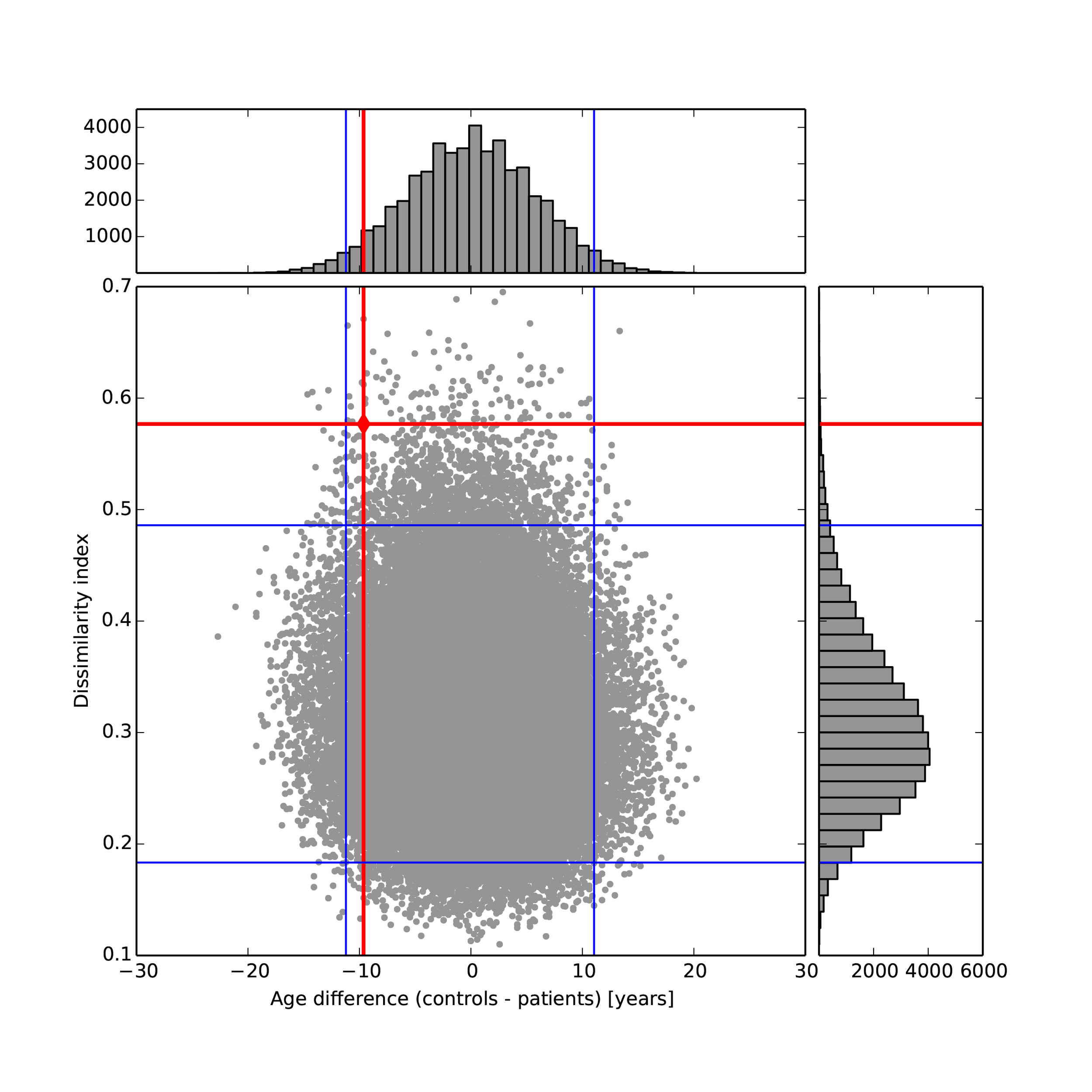

Supplement: Figure S1 — Dissimilarity as a function of the age-difference between controls and patients. Each of the points corresponds to one permutation. For each of the 50000 permutations the dissimilarity index and difference in age between the groups were determined. Their marginal distributions are displayed on top or at the right side, respectively. The red lines indicate the actual dissimilarity index (horizontal red line) and the actual difference in age (vertical red line). The blue lines indicate the 95-percentile range of the distributions. (TIF) [file pone.0110704.s001.tif]

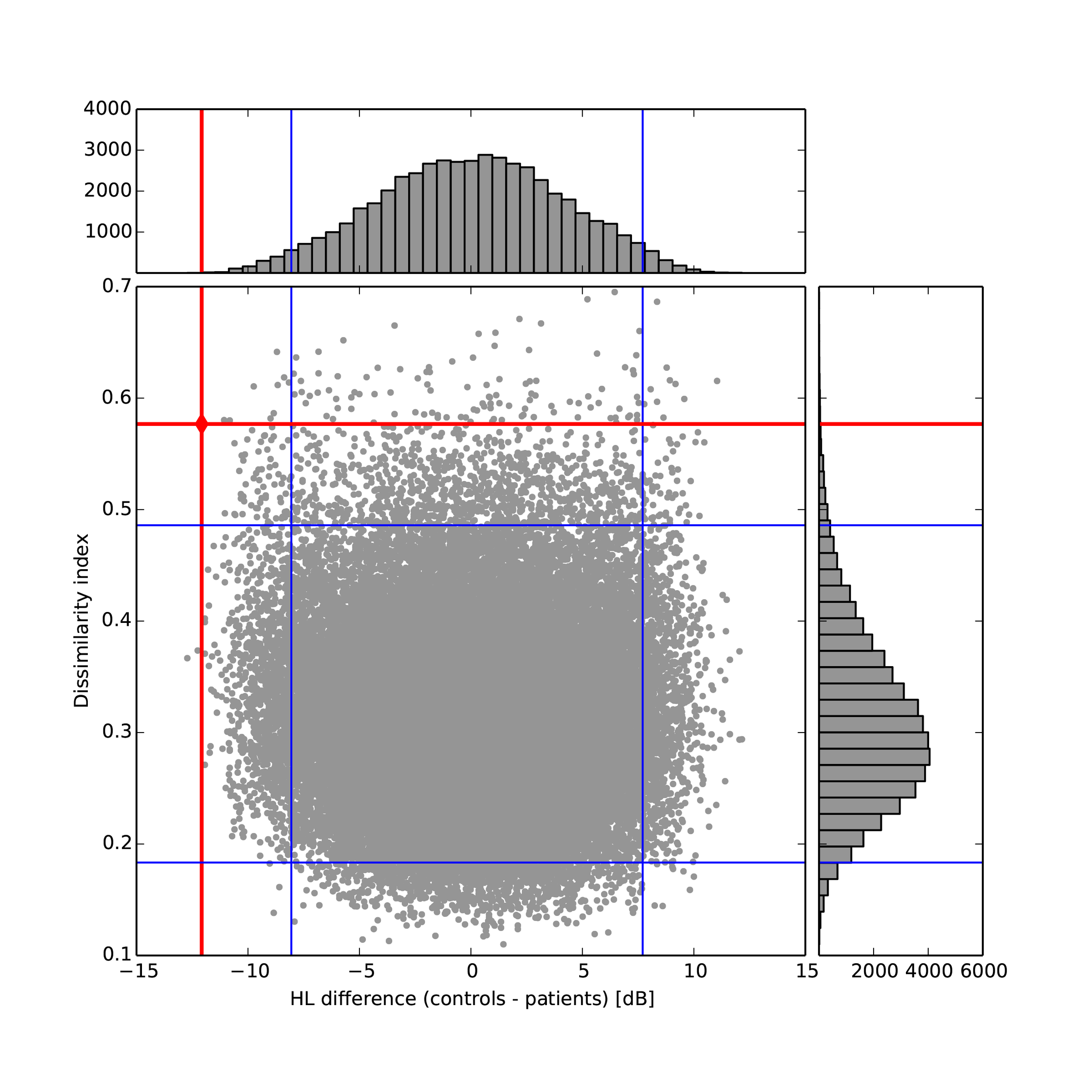

Supplement: Figure S2 — Dissimilarity as a function of the difference in hearing-level (HL) between controls and patients. For each of the 50000 permutations the dissimilarity index and difference in HL between the groups were determined. Their marginal distributions are displayed on top or at the right side, respectively. The red lines indicate the actual dissimilarity index (horizontal red line) and the actual difference in age (vertical red line). The blue lines indicate the 95-percentile range of the distributions. (TIF) [file pone.0110704.s002.tif]

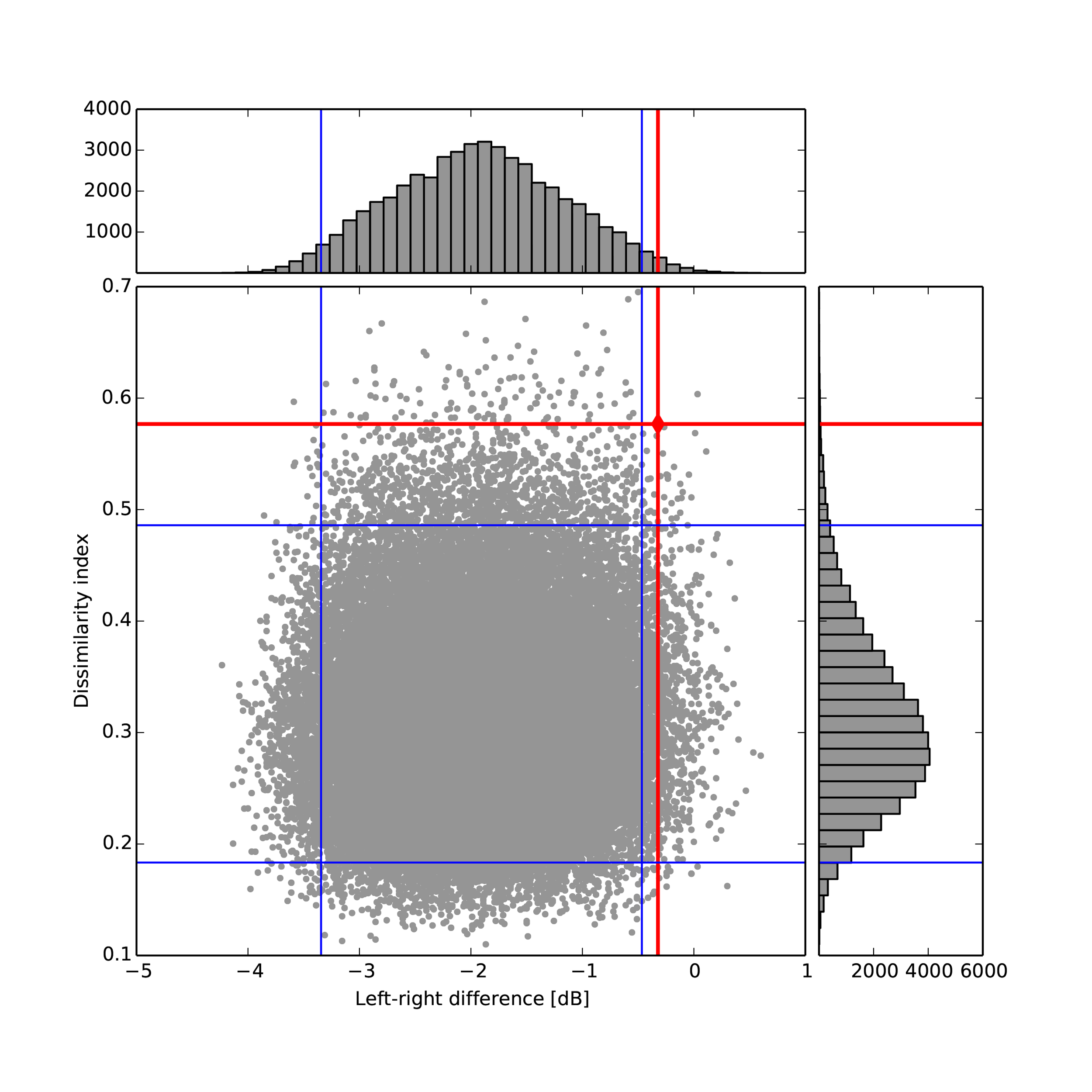

Supplement: Figure S3 — Dissimilarity as a function of the difference in left-right hearing-levels between controls and patients. For each of the 50000 permutations the dissimilarity index and difference in left-right hearing-levels between the groups were determined. Their marginal distributions are displayed on top or at the right side, respectively. The red lines indicate the actual dissimilarity index (horizontal red line) and the actual difference in age (vertical red line). The blue lines indicate the 95-percentile range of the distributions. (TIF) [file pone.0110704.s003.tif]

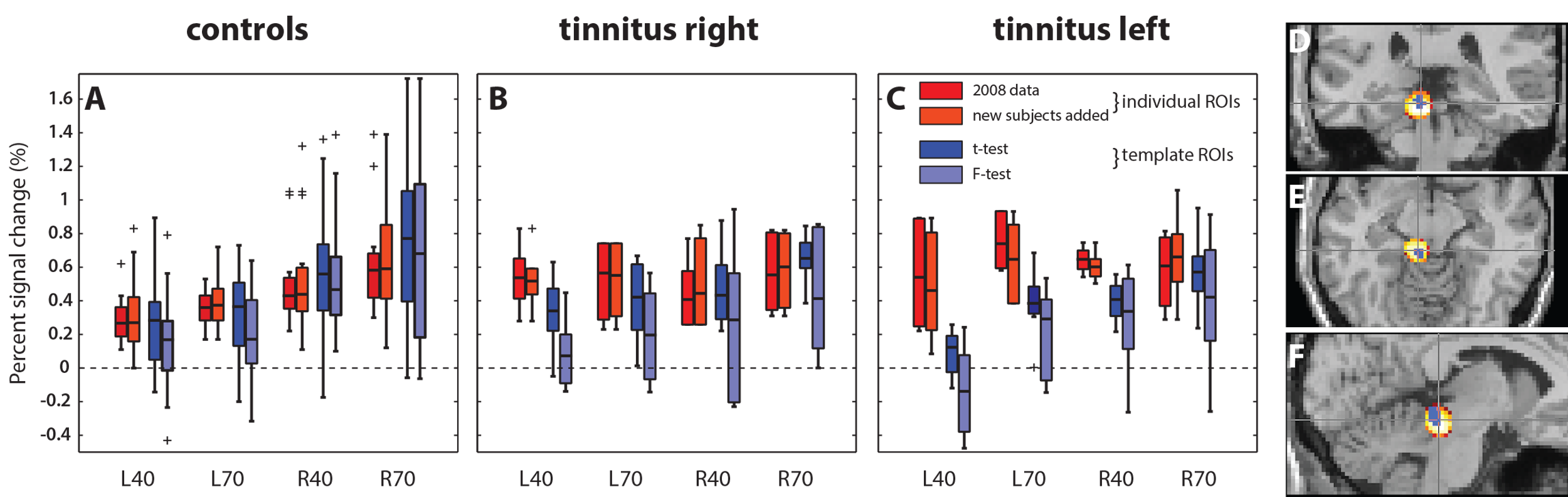

Supplement: Figure S4 — Sound-evoked responses in the left IC in controls (panel A), subjects with right-sided tinnitus (panel B) and subjects with left-sided tinnitus. The panels show four box plots per condition (left 40 dB, left 70 dB, right 40 dB, right 70 dB). The dark red bars show the average responses determined over the 10%most active voxels according to a (condition-wise) t-test in a manually drawn ROI. These results are identical to those reported by Lanting et al, (2008). The orange box plots represent results from identical analyses performed on the same subjects combined with the subjects that were added for this study (four controls, one patient with right-sided tinnitus and three with left-sided tinnitus; indicated with (*) in table 2). The dark blue box plots represent the analysis of the complete data-set with the identical t-test procedure again selecting the 10% most active voxel but using a ROI definition that was based on an anatomical template (MNI) and identical for each subject. The light blue box plots represent the results when an F-test (including all conditions) was used instead of the (condition-wise) t-test. The ROI selection has arguably the biggest effect on the size of the sound-evoked responses, especially in the patients groups. This effect is less dramatic in the controls. The insets (D–F) show the location and extent of the left IC ROIs. It shows a probability map, indicating the amount of overlap between subjects’ ROIs as used in the Lanting et al., 2008 study, thresholded at 80% overlap between all subjects in red-yellow colours. In blue is shown the template ROI as defined on the MNI template. (TIF) [file pone.0110704.s004.tif]
